# NOT JUST HOW MANY BUT WHO IS ON SHIFT: THE IMPACT OF WORKPLACE INCIVILITY AND BULLYING AMONG RCAS ON RESIDENT CARE

**DOI:** 10.1093/geroni/igz038.2725

**Published:** 2019-11-08

**Authors:** Heather A Cooke, Kaitlin Murray, Jennifer Baumbusch

**Affiliations:** 1 University of British Columbia, Vancouver, British Columbia, Canada; 2 Simon Fraser University, Vancouver, British Columbia, Canada

## Abstract

Much of the literature examining the link between care quality and staffing in long-term residential care focuses on staffing ratios and staffing mix; that is, how many staff are on shift. Far less attention has been devoted to exploring the impact of staff members’ workplace relationships, or who is on shift, on care quality. Of increasing concern is the potential for peer incivility and bullying to disrupt the respectful, collaborative and effective working relationships considered key to residential care aides’ (RCAs) care provision. This paper draws on data collected from a critical ethnography examining workplace incivility and bullying in a rural, not-for-profit care home. To date, more than 50 hours of participant observation, and 20 in-depth interviews with RCAs, licensed practical nurses, support staff, management and residents have been conducted. Thematic analyses identified three key themes: impact on resident safety; cutting corners; and impact on resident agitation and anxiety. Impact on resident safety highlights how incivility and bullying can result in non-adherence to two-person lift policies and procedures. Cutting corners outlines how RCAs’ relationships with their co-workers dictates to what extent they provide the requisite care to a resident for whom another RCA is responsible. Impact on resident agitation and anxiety focuses on residents’ reactions to the tensions that emerge between RCAs as a result of incivility and bullying. Findings highlight how peer incivility and bullying may indirectly influence certain quality indicators (e.g., pressure sores, psychotropic medication use) thereby offering additional insight into the staffing-care quality link.

